# The effects of mindfulness meditation versus CBT for anxiety on emotional distress and attitudes toward seeking mental health treatment: a semi-randomized trial

**DOI:** 10.1038/s41598-022-24256-9

**Published:** 2022-11-16

**Authors:** Daniela Aisenberg-Shafran, Liav Shturm

**Affiliations:** Department of Clinical Psychology of Adulthood and Aging, Ruppin Academic Center, Emek Hefer, Israel

**Keywords:** Geriatrics, Human behaviour

## Abstract

Older adults often avoid seeking psychological treatment, thus challenging their ability to cope effectively with anxiety, stress, and depression. The current study aimed to compare the effects of a mindfulness intervention with cognitive behavioral therapy (CBT) on measures of emotional distress and attitudes toward seeking mental health treatment among older adults. Twenty-four seniors were assigned to three groups: (1) Mindfulness-based intervention for seniors (MBIS), (2) CBT for anxiety, or (3) a care-as-usual control group. Participants in the two intervention groups of eight weekly sessions were randomly assigned. Results showed that attitudes toward seeking psychological treatment and depression, anxiety, and worry levels were evaluated before and after the interventions. Following both interventions, participants considered the prospect of utilizing psychological treatment more positively, whereas no changes were observed in the control group. Interestingly, worry levels were eased only in the MBIS group, and anxiety levels were eased only in the CBT group. Our findings support the understanding that cognitive group interventions can effectively achieve their intended aim (decreasing worry or anxiety) and positively impact attitudes toward psychological treatment.

*Trial registration*: clinicaltrials, NCT04165005, 15/11/19.

## Introduction

Aging can be experienced as a complex and challenging process, considering the increased exposure and proximity to death alongside the physical, emotional, and cognitive decline characterizing old age. Thus, interventions are critically needed to improve the elderly population's quality of life and ease its pain. Such interventions must be made accessible, as individuals in this age group often rebuff recommendations to utilize them^1^. The current study will examine the influence of a mindfulness-based intervention for seniors (MBIS) and a cognitive behavioral therapy (CBT) intervention on various psychological measures and seniors' attitudes toward seeking mental health treatment.

Mindfulness has been defined in several ways, all sourced in the Buddhist tradition. The most accepted Western definition defines mindfulness as the maintenance of prolonged attention on human experiences (such as thoughts, emotions, and bodily sensations) in the present, "from moment to moment," and in an accepting, non-judgmental manner^2^. This definition incorporates three components that are also referenced in the bulk of Buddhist literature on the topic of mindfulness: (1) attentive awareness; (2) current experience, and (3) acceptance^3^.

The past 30 years have been marked by extensive literature pointing to the significant advantages of meditation on various factors related to mental and physical health^4,5^. The first successful studies on mindfulness examined patients suffering from chronic pain and tension. These first studies encouraged the development of mindfulness-based stress reduction (MBSR) for this population^6^. At a later stage, mindfulness-based cognitive therapy (MBCT) was developed. MBCT is a therapeutic intervention based on MBSR. However, it differs from it in its focus on depression and in its use of a dominant psycho-educational feature that focuses on the prevention of relapse^7^. These two standard interventions comprise eight weekly sessions averaging 2.5 h. A meta-analysis of 64 studies found that mindfulness-based interventions are effective in coping with chronic pain, fibromyalgia, coronary artery diseases, cancer, anxiety disorders, depression, and contextual stressors^8^.

Several theoretical underpinnings have been suggested to explain the mechanism behind the psychological-therapeutic effect accompanying the methodological practice of mindfulness. These theoretical explanations have gained broad empirical support from meta-analyses on meditation research^9^. One theoretical explanation suggests that focusing attention and responding with acceptance and non-judgment regarding life experiences improves attentional and self-regulatory capacities. Another explanation is that the suggestion to accept any thoughts and emotions without avoidance encourages a diminished use of repression and assists in arousing autobiographical memories from specific places and times, both of which are of considerable value to mental health treatment. A third theoretical explanation suggests that mindfulness helps diminish negative ruminative thinking by bringing negative thoughts to consciousness and decreasing reactivity to them. Mindfulness also appears to influence emotional and cognitive flexibility and self-perception changes.

The current study aimed to expand our understanding and knowledge of mental health in old age and to highlight its importance. The senior population is continuously challenged with maintaining and advancing its well-being. Data from a U.S. Census study that included 12,312 adults 64 years of age or older suggested that 11.4% of older adults cope with anxiety disorders and 6.8% cope with emotional disorders, all of which met the clinical criteria for diagnosis^10^. It is important to note that by including symptoms outside the clinical criteria for diagnosis, the percentage of depression and anxiety increases significantly in the elderly, more than in any other age group^11^.

Anxiety and the decline of one's physical state are cyclically associated. Anxiety encourages avoidance behavior, which promotes muscle weakening and pathology of the muscular and skeletal systems, which then foments further atrophy^12^. A meta-analysis on loneliness in old age^13^ identified general themes explaining and detailing the experience of loneliness in old age: the loss of significant interpersonal relationships, the negative influence of loneliness on self-perceptions, and negative emotions associated with loneliness, such as anxiety, sadness, and anger. These themes were found to be reciprocally associated with one another. The biopsychosocial model^14^ describes the mutual influence of the individual's physical, mental, and social state, positing a more significant interrelation in the elderly than in younger individuals. This model suggests that a chronically stressful environment significantly increases vulnerability to illness and the loss of functional capacities, which, in turn, increase anxiety and a sense of helplessness. Another example relates to chronic illnesses in old age, such as anxiety, depression, and bipolar disorder, which are all associated with a higher risk for suicidal behavior in old age. Aligning with Garroway and Rybarczyk's model, depression and anxiety were found to encourage cognitive biases that act as psychological barriers which potentially delay or prevent adaptive behavior in response to challenges in old age. For example, elderly persons may eschew the use of a walking stick, as it symbolizes a deteriorating physical state and some level of submission––experienced as symbolic threats to one's identity and ego.

The increased vulnerability to emotional distress in old age highlights the critical need for effective short-term interventions that can quickly improve seniors' health and quality of life. A study on elderly participants that examined the efficacy of a brief, 4-week behavioral intervention found significant improvement in various psychological and cognitive measures^15^. Another study examined a brief 4-week behavioral intervention intended to assist seniors with chronic sleep difficulties and found significant improvement in the sleep quality of the participants in the intervention group^16^.

Only a few research studies have examined the influence of mindfulness on measures of anxiety and depression in the elderly. For example, a meta-analysis examining existing mindfulness-based interventions and their influence on mental and physical well-being in the elderly included only four studies^17^. Two of these studies examined the influence of a standard, 8-week MBCT intervention, finding decreases in anxiety and depression measures with a moderate-to-high effect size^18,19^. The other two studies examined a standard 8-week MBSR intervention, finding a decrease in measures of anxiety and depression with a high effect size^20,21^. None of these four studies compared the experimental group with a control group that underwent a different treatment intervention. Three of the studies offered no comparison between the experimental group and a control group. An MBCT study on elderly individuals^22^, incorporating an activity-based control treatment group and whose experimental group was exposed to a shortened intervention (1.5 h), also found a decrease in measures of anxiety and had a high effect size. Although these interventions demonstrated their efficacy, the various interventions were characterized by high attrition rates (about 15%). The primary sources of these attrition rates were gender (men discontinued their participation more than women), a few years of education, expectations for a quick change, and high levels of distress^23,24^. A meta-analysis by Carmody and Baer^25^ suggested a solution for these high attrition rates, which examined 25 studies that implemented mindfulness-based interventions of various lengths and age groups. They believed that the high number of sessions made perseverance during the sessions problematic. They did not offer any evidence to suggest that abridged versions of mindfulness interventions are less effective than the standard intervention in diminishing emotional distress; however, they suggested shortening mindfulness interventions to make them more accessible for population groups (i.e., the elderly) that have difficulties committing to standard interventions.

Another popular intervention for depression and anxiety in the elderly is CBT. As its name suggests, CBT aims to modify the thoughts and behaviors that cause distress to the patient while cultivating thoughts and behaviors that are more effective for the patient. CBT's' basic assumption is that emotional distress results from how individuals perceive and interpret their reality and is not necessarily the consequence of objective reality. CBT, therefore, places significant emphasis on solving current issues^26^. A review of multiple meta-analyses examining the efficacy of CBT for anxiety and depression in the elderly found significant efficacy, as displayed by the higher than average effect sizes for CBT^27^.

It is important to note that alongside the many benefits of these interventions and the many and varied solutions mental health treatment offers the elderly, this population is less likely to seek mental health services than younger age groups^1^. Three factors were found to have the most significant impact on elderly persons' attitudes toward seeking treatment: (1) their perception of their aging process, (2) their perception of psychologists, and (3) their perception of social support. Furthermore, stigma––a distinguishing characteristic or trait that disqualifies those that carry it from full social acceptance^28^––is one of the central factors for why the elderly avoid seeking mental health treatment^29^.

Considering elderly persons' distancing themselves from mental health services, the need for short-term interventions to help modify the cognitive perceptions associated with attitudes toward seeking mental health treatment becomes apparent. The appropriate intervention should contribute to increasing the utilization of therapy by the elderly, whether individual or group therapy. As noted, mindfulness-based interventions assist individuals in accepting their current thoughts and emotions non-judgmentally. In this way, the mindfulness-practicing individual observes or contemplates his thoughts instead of giving vent to an emotional reaction to them. Introspection helps create mental and emotional distance between the individual and their thoughts, facilitating, among other things, a more flexible approach to the individual's thoughts, including non-adaptive perceptions.

The current study aimed to contribute to the existing literature in two ways: (1) Establishing support for a short-term mindfulness-based intervention for seniors to be effective in reducing age-related anxiety and depression and encouraging seniors' utilization of mental health treatment; (2) Comparing a mindfulness-based intervention with an alternative treatment intervention (CBT).

Mindfulness-based interventions that have been demonstrated to reduce anxiety and stress in the elderly often include breathing exercises. Further, behavioral relaxation techniques such as diaphragmatic breathing are most effective in reducing anxiety and stress in the elderly^21^. To our knowledge, research has yet to examine the influence of mindfulness on anxiety in the elderly relative to a control group that isolates the effects of diaphragmatic breathing (a potential confounding variable). For this reason, it is critical to isolate and examine the unique attentional component of mindfulness-based interventions.

Similarly, to our knowledge, research has yet to examine the influence of mindfulness on attitudes toward seeking mental health treatment among the elderly. Thus, another practical goal of this study was to examine an easily deliverable and accessible group intervention in a nursing home. In the wake of the COVID-19 crisis, it has become clear that efforts should be made to bring therapy to where the people are and not rely on inviting them to professional centers^30^.

Research hypotheses

1. An interaction between group and time on the measure of emotional distress will be found: Mindfulness-based intervention for seniors (MBIS) and cognitive behavior therapy (CBT) groups will significantly improve elderly participants' anxiety and depression compared with a control group.
2. An interaction between group and time on the measure of attitudes toward seeking mental health treatment will be found: Specifically, an MBIS group will significantly improve participants' attitudes toward seeking mental health treatment compared with a control group. We did not posit the direction of the CBT group in this interaction.

## Method

### Participants

Twenty-four elderly persons with normative functioning participated in this study. We used G*power software to determine the number of participants, setting the analysis to ANOVA: repeated measures, within-between interactions, expecting an effect size of 0.25, with three groups. All participants (*N* = 22) were recruited from a senior's club in the center of Israel at the end of 2019 (all participants were women; *M*age = 73.12; *SD* = 5.67). Participants had been invited through a brief lecture and explanation of the study to join a group activity (not a psychological intervention). Participation in the study was voluntary as part of the nursing home's enrichment program. Written informed consent was obtained from all participants included in the study. Inclusion criteria comprised membership in the nursing home, aged 65 or older, and scoring 24 and above on the Mini-Mental State Examination^31^.

The participants were assigned to three groups (MBIS, CBT, and a care-as-usual control group), with eight participants in each group. Of the 12 participants who began the MBIS course, three canceled their participation (25% disengagement), and one discontinued her participation after the second session (10% attrition rate). Of the 12 participants who began the CBT course, two canceled their participation (16% disengagement), one ended her participation after the second session, and a fourth ended her participation after the third session (20%attrition rate). Reasons for attrition were an unexpected move to a different facility, difficulty committing, lack of interest, and a decline in physical-mental health. A make-up session was provided to participants who missed the first session.

### Procedure

At the study's recruitment stage, an informational session took place at the nursing home, during which a brief general explanation was provided, participation was encouraged, and, at its culmination, those interested signed up. An additional session was scheduled for those who agreed to participate in the study. At this additional session, prospective participants signed informed consent forms for the study, completed a demographic questionnaire, a questionnaire tapping attitudes toward seeking mental health treatment, and scales measuring emotional distress (Time 1; *see* Tools).

At the end of the additional session, participants were assigned to the three groups according to the following stages. Participants were randomly assigned (using random lists created in Excel with participants' ID) to one of two intervention groups: (1) an abbreviated mindfulness-based intervention for seniors (MBIS); (2) cognitive behavioral therapy (CBT). Those agreeing to participate but could not commit to all sessions were assigned to a (3) care-as-usual control group, which attended the regular activities in the facility and performed the experimental meetings before and after the interventions of the first two groups. The MBIS and the CBT intervention sessions lasted 30 min and met for eight weekly sessions during January and February 2019. The interventions were conducted in a designated room in the nursing home and administered by an instructor with two years of experience practicing mindfulness meditation. At the conclusion of the 8-week course, all participants completed the questionnaire battery for the second time (Time 2). The data collection at Time 2 was performed by a research assistant who was blind to the study conditions and did not have access to study information.

### Ethical approval

Research involving human participants and/or animals: We confirm that the study was approved by the institutional and Helsinki ethics committee (Ruppin IRB and Beer-Yaakov hospital) and certify that the study was performed in accordance with the ethical standards as laid down in the 1964 Declaration of Helsinki and its later amendments or comparable ethical standards. Participants received an explanation about the study and signed informed consent before committing to the study. Trial registration: clinicaltrials, NCT04165005, 15/11/19.

## Tools

### Intervention protocols

#### Mindfulness-based intervention for seniors (MBIS)

The MBIS course is based on the standard MBCT and MBSR courses (see Appendix 1 for the full intervention program). The course program was designed applying the principles of Hölzel et al.'s^32^ model. Each 30-min session included practice (10–20 min), psycho-education (5–10 min) or sharing of experiences (3–10 min), or both. In addition, all participants were asked to practice for 10 min daily, repeating the exercise that had been demonstrated and practiced in the session. During the sessions, homework completion was verified at every meeting to ensure compliance, and participants were invited to share their experiences after every exercise. Aside from the formal practice, participants were instructed to integrate attention activities into their daily activities, according to their choice. Aligning with Hölzel et al.'s^32^ model, the course began with demonstrations of attention regulation and body awareness exercises. Later, other exercises were added that activate the processes of emotion regulation and change in perspective of the self. The abbreviated version of the MBIS is well suited for seniors due to its length, fewer requirements, and that it can be administered by instructors that are not required to undergo lengthy and expensive training.

#### *Cognitive behavior therapy (CBT)* *for anxiety*

The CBT course is based on the manual for CBT (abbreviated) therapists^33^. The course comprised eight sessions, 30 min each (see Appendix 2 for the full intervention program). Each session included psycho-education and a joint discussion (20 min), a diaphragmatic breathing exercise (5 min), and an explanation of the home exercise (5 min). The home exercise took 10 min, parallel with the exercise practiced during the session. Both interventions began at the nursing home approximately a week after the participants completed the questionnaires (Time 1).

### Study questionnaires: outcome measures

Anxiety was measured by two questionnaires: (1) The Beck Anxiety Inventory (BAI) is a 21-item multiple-choice questionnaire that measures anxiety severity. Each item offers four response options, ranging from 0 to 3. Total questionnaire scores ranged from 0 to 63, with high total scores indicating high anxiety. The BAI has high internal reliability (*α* = 0.92) and has been validated^34^, and (2) The Penn State Worry Questionnaire (PSWQ) is a 16-item multiple-choice questionnaire that measures symptoms of worry. Each item has five response options, with scores ranging from 1 to 5. High total questionnaire scores indicate high levels of worry (total questionnaire scores range from 16 to 80). The PSWQ has high internal reliability (*α* = 0.91) and has been validated^35^.

Depression was measured with the Patient Health Questionnaire-9 (PHQ-9). The 9-item PHQ-9 is a multiple-choice questionnaire that measures symptoms of depression. Each item offers four response options, with scores ranging from 0 to 3, with high scores indicating high levels of depression. This questionnaire has high internal reliability (*α* = 0.89) and has been validated^36^.

Inventory of Attitudes Toward Seeking Mental Health Services (IASMHS). The 24-item IASMHS taps three factors: Psychological Openness, Help-Seeking Propensity, and Indifference to Stigma. Each item offers five response options, with scores ranging from 1 to 5. Higher scores indicated a greater inclination to seek mental health treatment (total questionnaire scores range from 24 to 120). This questionnaire has high internal reliability (*α* = 0.87) and has been validated^37^.

## Results

### Demographic variables

A one-factor analysis of variance was conducted to compare the two intervention groups via the following variables: chronological age, subjective age, years of education, socioeconomic status (SES), frequency of contact with grandchildren, and time spent socially. A χ2 test was also calculated to compare the two intervention groups via gender, living arrangement, type of residence, and familial status (see Table 1). There were no significant differences, aside from years of education, between the two groups. A further analysis using the Scheffé criterion for significance found that the MBIS group had significantly fewer years of education than the control group, *F*(2,21) = 5.86, *p* < 0.01).

**Table 1:** Means (*SD*s) and differences between groups in demographic variables.

	MBIS (*n* = 8)	CBT (*n* = 8)	Control (*n* = 8)	All Participants (*N* = 24)	Statistical parameter	*p* <
Gender (number of women)	6	8	8	22	X^2^ = 4.36	–
Age	76.38 (5.24)	72 (7.09)	71 (3.02)	73.13 (5.67)	*F* = 2.26	–
Change in subjective age*	0.63 (10.16)	0.81 (9.54)	0 (0)	0.48 (7.69)	*F* < 1	–
Years of Education	10.50 (2.78)^(−)^	12.25 (1.98)	14.25 (1.67)^(−)^	12.33 (2.62)	*F* = 5.86	0.01
SES	3.38 (0.74)	3.38 (0.74)	2.88 (0.84)	3.21 (0.78)	*F* = 1.16	–
Contact with grandchildren	5.13 (0.64)	4.38 (1.19)	4 (1.6)	4.5 (1.25)	*F* = 1.79	–
Time spent socially	4.88 (0.35)	4.75 (0.46)	4.75 (0.7)	4.79 (0.51)	*F* < 1	–

(−/ +) Reflect significant differences between the groups in follow-up analyses.

*Subjective age refers to participants' responses about how old they feel; the discrepancy between chronological age and subjective age is recorded in years (for more details, see^38^).

### Correlations between research measures at time 1 and the demographic variables

Pearson correlations were calculated to examine associations between the research measures at Time 1 and the demographic variables (see Table 2). Time spent socially was moderately negative and significantly correlated with depression (*r* = −0.53, *p* < 0.05) and with anxiety (*r* = −59, *p* < 0.01). Thus, more time spent socially was associated with lower depression and anxiety. Furthermore, the measure of worry was moderately positive and significantly correlated with anxiety (*r* = 0.523, *p* < 0.01) and depression (*r* = 0.6, *p* < 0.01). Thus, more worry was associated with greater anxiety and depression. A strong positive correlation between anxiety and depression was also found (*r* = 0.72,*p* < 0.01). In other words, higher anxiety levels correlated with higher levels of depression. Finally, a moderately negative and significant correlation was found between attitudes toward seeking mental health treatment and frequency of contact with grandchildren (*r* = −0.5, *p* < 0.05). Thus, more frequent contact with grandchildren was associated with a less negative perception of mental health treatment.

**Table 2:** Correlations between research variables at Time 1 and the demographic variables.

	Age	Years of education	SES	Frequency of contact with grandchildren	Time spent socially	Attitudes -seeking MH treatment T1	Depression time 1	Anxiety time 1	Worry time 1
Age	1	−0.296	−0.114	0.267	−0.051	−0.259	−0.184	0.053	−0.179
Years of education		1	−0.228	−0.213	0.25	−0.019	−0.306	−0.158	−0.181
SES			1	−0.067	0.005	0.222	−0.233	−0.556**	−0.173
Frequency of contact with grandchildren				1	0.034	−0.502*	0.067	0.118	−0.083
Time spent socially					1	0.024	−0.532**	−0.586**	−0.189
Attitudes re: seeking MH treatment, time 1						1	−0.255	−0.222	−0.09
Depression, time 1							1	.722**	.600**
Anxiety, time 1								1	.523**
Worry, time 1									1

*p < .05, **p < .01.

### Measures of emotional distress and attitudes toward seeking mental health treatment (BAI, PSWQ, PHQ-9, IASMHS)

**Figure 1:**
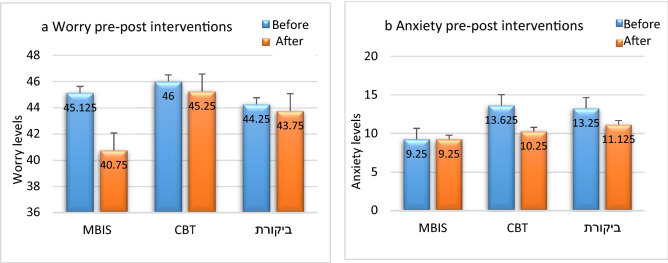
(**a**) Worry and (**b**) anxiety levels before and after the interventions.

A mixed-design repeated-measures ANOVA was conducted to examine emotional distress and attitudes toward seeking mental health treatment as a function of time (before/after intervention) as a within-subject variable and intervention type (MBIS/CBT/control) as a between-subject variable (see Table 3 for means and *SD*). A marginally significant main effect was found for time on the anxiety measure (BAI), *F*(1,21) = 3.22, *p* < 0.09, n2p = 0.13. In contrast to the research hypothesis, no interaction was found between time and group. However, aligning with the a-priori hypotheses, a follow-up analysis comparing the CBT group and the other two groups' anxiety measures showed a marginally significant effect, *F*(1,21) = 3.63, *p* < 0.08, n2p = 0.15. Thus, the CBT group reported a greater decrease in anxiety levels than the MBIS and control participants (see Fig. 1 and Table 4).

**Table 3:** Means (and SDs) for IASMHS, BAI, PSWQ, PHQ-9.

	MBIS (*n* = 8)	Before	(Time 1)	All participants (*n* = 24)	MBIS (*n* = 8)	After	(Time 2)	All participants (*N* = 24)
		CBT (*n* = 8)	Control (*n* = 8)			CBT (*n* = 8)	Control (*n* = 8)	
IASMHS	79.63(7.54)	84.5(8.91)	84.38(6.63)	82.83(7.76)	85.13(9.99)	90.38(9.7)	83.88(7.47)	86.46(9.18)
BAI	9.25(7.29)	13.63(5.55)	13.25(16.8)	12.04(10.75)	9.25(6.5)	10.25(5.75)	11.13(13.75)	10.21(9)
PSWQ	45.13(11.9)	46(11.61)	44.25(12.44)	45.13(11.48)	40.75(10.77)	45.25(11.13)	43.75(11.85)	43.25(10.93)
PHQ-9	5.75(3.45)	7.13(5.19)	6(6.23)	6.29(4.9)	5.38(3.78)	5.25(4.56)	6.25(5.55)	5.63(4.5)

**Table 4:** Effects for IASMHS, BAI, PSWQ, PHQ-9.

	Effects	Meaning	*df*	*F*	*p* <	*n*^*2*^_*p*_
IASMHS	Time	The attitude toward seeking mental health treatment measure was higher at Time 2 than at Time 1 for all groups	1,21	14.07	.01	.4
	Time x Group			4.57	.05	.3
	Contrast	The attitude toward seeking mental health treatment measure was higher for the MBIS and CBT groups than for the control group at Time 1 and Time 2		9.1	.01	.3
BAI	Time	The anxiety measure was lower at Time 2 than at Time 1 for all groups	1,121	3.22	.09	.13
	Contrast	The anxiety measure was lower in the CBT and control groups than in the MBIS group at Time 1 and Time 2		3.63	.08	.15
PSWQ	Time	The worry measure was lower at Time 2 than at Time 1 for all groups	1,21	4.93	.04	.19
	Contrast	The worry measure was lower in the MBIS group than in the CBT and control groups		4.38	.05	.17
PHQ-9	All Effects		1,21	< 1.11	Not Measured	

Regarding the worry measure (PSWQ), aligning with the research hypothesis, a main effect was found for time, *F*(1,21) = 4.93, *p* < 0.04, such that worry decreased between Time 1 and Time 2. Also, for this measure, no interaction was found between time and group, contrary to the research hypothesis. However, aligning with the a-priori hypotheses, a follow-up analysis comparing the MBIS group and the other two groups showed a significant effect,*F*(1,21) = 4.38, *p* < 0.05. Thus, the MBIS group reported a greater decrease in worry levels than the CBT and control participants.

**Figure 2:**
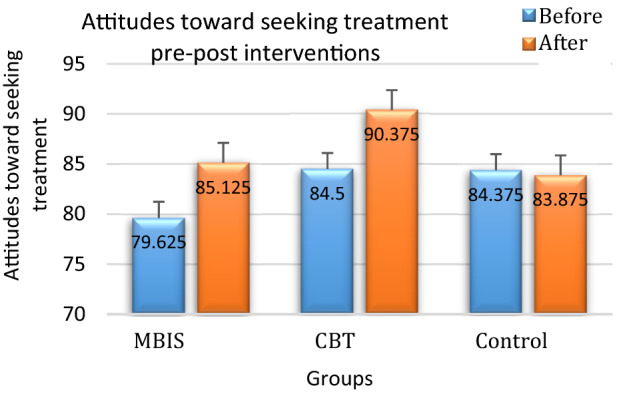
Attitudes toward seeking mental health treatment before and after the intervention.

Lastly, and in contradiction to the research hypothesis, no effects were found for depression (PHQ-9). Regarding attitudes toward seeking mental health treatment (IASMHS), a main effect for time was found, *F*(1,21) = 14.07, *p* < 0.01, such that all participants reported more positive attitudes toward seeking mental health treatment at Time 2. Most importantly, a significant interaction was found between time and group, yielding a moderate effect size, *F*(1,21) = 4.57, *p* < 0.05, n2p = 0.3. A follow-up analysis showed a significant difference between the improvement in attitudes toward seeking mental health treatment in the MBIS and CBT group and the control group, *F*(1,21) = 9.1, *p* < 0.01. In other words, all participants in the intervention groups reported more positive attitudes regarding mental health treatment after the intervention than the control group participants, who showed no change (see Fig. 2 and Table 4).

### Analysis of measures before and after the intervention

Pearson correlations were calculated to analyze the research measures before and after the interventions (see Table 5). A moderately positive and significant correlation was found between change in subjective age and years of education (*r* = 0.47, *p* < 0.05): the fewer the years of education, the greater the difference in subjective age from Time 1 to Time 2, and the younger the perceived age from Time 1 to Time 2. Thus, the less educated the elderly participant, the younger they reported feeling after the intervention. In addition, a moderately positive and significant correlation was found between change in worry and years of education (*r* = 0.41, *p* < 0.05). In other words, more years of education were associated with a greater difference in the worry measure from Time 1 to Time 2. Furthermore, a moderately negative and significant correlation was found between changes in attitudes toward seeking mental health treatment and years of education (*r* = −0.43, *p* < 0.05). In other words, the fewer the years of education, the more the attitude toward seeking mental health treatment improved from Time 1 to Time 2. As noted, the groups were not uniform in education level. The study findings regarding age will be further addressed in the Discussion.

**Table 5:** Analysis of measures before and after the interventions.

	Change in subjective age	Change in anxiety	Change in worry	Change in depression	Change in attitude	Age	Years of educa- tion	SES	Frequency of contact with grandchildren	Time spent socially
Change in subjective age	1	−0.111	0.067	−0.091	−0.111	−0.1	.467*	−0.162	−0.132	0.138
Change in anxiety		1	0.275	0.156	0.065	−0.002	−0.044	0.27	−0.223	0.237
Change in worry			1	−0.003	−0.147	0.038	0.413*	−0.085	−0.332	−0.027
Change in depression				1	−0.427*	0.2	0.334	−0.066	0.056	0.211
Change in attitudes					1	0.295	− 0.426*	0.339	0.01	−0.03
Age						1	−0.296	−0.114	0.267	−0.051
Years of education							1	−0.228	−0.213	0.25
SES								1	−0.067	0.005
Frequency of contact with grandchildren									1	0.034
Time spent socially										1

*p < .05, **p < .01.

Lastly, a moderately negative and significant correlation was found between a change in attitude toward seeking mental health treatment and a change in depression (*r* = −0.43, *p* < 0.05). Thus, as depression decreased from Time 1 to Time 2, attitude toward seeking mental health treatment improved from Time 1 to Time 2.

## Discussion

The purpose of the current study was to examine the effects of a brief mindfulness intervention adapted to elderly adults on measures of emotional distress (i.e., anxiety, depression, and worry) and attitude toward seeking mental health treatment among healthy seniors. The primary findings suggest that the MBIS intervention engenders a positive change in diminishing feelings of worry, whereas the anxiety-focused CBT intervention successfully improved anxiety symptoms. No improvement was found in the depression measures in any of the study groups. Regarding attitudes toward seeking mental health treatment, participants in the MBIS and the CBT interventions reported a more positive attitude regarding seeking mental health treatment, whereas those in the control group reported no change in attitude.

As noted, the variable of participants' years of education yielded significant associations with change in subjective age, worry, and attitude toward seeking mental health treatment as a result of the interventions. These associations suggest that having more years of education is linked to diminished mental health and a negative view of therapy. Whereas these correlations are surprising, they are likely the result of between-group differences in this variable: Years of education in the control group were higher than in the other groups and significantly higher than in the MBIS group. In other words, the control group was characterized by its relative abundance of years of education, alongside its other defining characteristic––receiving no intervention. The discrepancy in years of education between the control and CBT groups was insignificant. However, as the interactions between the two intervention groups and worry and anxiety across time were significant, the intervention effect may be understood to be sustainable. In other words, it is less likely that the MBIS group's fewer years of education explains the differences between the control and two intervention groups. Moreover, it is likely that the increased change in subjective age and the younger perception of the intervention groups from Time 1 to Time 2, as well as the acceptance that characterizes MBIS, are the consequence of the interventions rather than years of education.

The variable influence of the MBIS and CBT interventions on the measures of worry and anxiety warrants close attention. Notably, only the MBIS group improved in the worry measure, whereas only the CBT group improved in the anxiety measure. Many mindfulness-based interventions reported in the literature, however, suggest their distinct effectiveness (via moderate-high effect sizes) on both anxiety and depression (for a comprehensive meta-analysis, see^39^). A possible explanation for this pattern of findings relates to the therapeutic mechanism at the core of these interventions: whereas CBT is a symptom-focused intervention aiming to directly diminish the targeted symptom over a short period^26^, mindfulness-based interventions focus on accepting the symptom and attending to it openly and non-judgmentally^2^. Interventions of the mindfulness type are process-focused and require participants to perform more practice to enhance their effectiveness^40^. Thus, the lack of a significant decrease in anxiety in the MBIS group may be because the applied study model mandated very little practice. As the MBIS group performed their homework assignments only partially, this helps substantiate the noted explanation. The decrease in worry in the MBIS group can be explained by its inherent focus on learning to introspect and openly accept a range of experiences, including worry.

In contrast to the research hypothesis, no significant decrease in levels of depression was revealed in the MBIS group. An explanation for the lack of significant improvement in depressive symptoms after the intervention may be tied to a "floor effect" characteristic of a relatively healthy sample: The relatively low scores on the PHQ-9 questionnaire (as a measure of depressive symptoms; *see* Table 3) may be a consequence of its narrow scale (0–27), and the fact that the sample was not a clinical population may have diminished the prospect of a significant effect. However, aligning with the research literature^41^, more time spent socially was linked to lower levels of depression and anxiety at Time 1.

In both intervention groups, the attitude toward seeking mental health treatment was more positive after the interventions. One can assume that this positive change in both groups is because both interventions incorporated an open and non-judgmental discussion of negative emotions. Thus, it may be that the stigma associated with mental health treatment was diminished among the intervention participants. Likewise, it is likely that because the administrator was a clinical psychology student (and presented himself as such), the relationship with him contributed to the participants' enhanced perception of psychologists––a factor significant in improving attitudes toward seeking mental health treatment^1^. Interestingly, whereas levels of depression did not decrease significantly overall, a decrease in depression levels was found to be associated with an improvement in attitudes toward seeking mental health treatment. As the positive influence of mindfulness practice on depression has been frequently replicated^17^, it would be helpful to examine whether attitudes toward seeking mental health treatment are influenced by mindfulness practice, mediated by levels of depression.

A noteworthy finding in this study is that a higher frequency of contact with grandchildren was associated with negative attitudes toward seeking mental health treatment. In seeking to understand this finding, we can suggest that more frequent contact with grandchildren is associated with better mental health^42^, thus minimizing the need to seek mental health treatment or even think about it. This aligns with our finding that time spent socially (not only with grandchildren) was negatively correlated with anxiety and depression, likely enhancing mental health.

The current study has several strengths as a pre-post design with two types of interventions and a control group. Nevertheless, several significant limitations should be noted. First, a small sample affected the statistical strength of the data analyses. Many efforts were made to encourage participants' perseverance in the study and avert attrition, starting with a potential selection bias of participants, allowing those who can commit to attending all sessions. Efforts were also obtained in groups and personal reminders sent, and alternative session dates were offered when the originally scheduled date meant limited participation. Among the participants who committed to the interventions, only one withdrew in one group and only two in the other group, all at the beginning. This is an improvement compared to previous studies that showed attrition after long effort and engagement. Further research should retain the intimate nature of group size to maintain the efficacy of these interventions for older adults^43,44^. However, the number of groups should be increased to boost the statistical strength of the analyses. Secondly, the enrichment room at the nursing home was not a sterile or ideal setting, as it was subject to external noise that may have distracted or disturbed the participants. In addition, the participants' personal limitations associated with their age (e.g., partial hearing loss, attention, or reading difficulties) may have challenged them to absorb some of the content or engage in practice and learning. These limitations likely weakened the various effect sizes. To avoid attrition, the study researchers were relatively lenient regarding homework assignments and follow-ups.

This study did not measure mindfulness using a mindfulness questionnaire, meaning that there was no manipulation check performed in this study. These leniencies encouraged participants to continue participation in the course but weakened the intervention fidelity. Third, to adapt the interventions to the elderly participants, this study shortened the session length in the MBIS intervention to ease the course's intensiveness without impairing the intervention's essential features. However, as elderly individuals have been reported to benefit greatly from consistent and frequent practice^45^, it would be helpful to examine the efficacy of a research design incorporating home assignments performed at a group level. Home assignments performed in a group setting should encourage better compliance and more frequent practice, thus more successfully implementing the intervention^40^. Feasibility studies are also needed to support the effectiveness of the MBIS, as it is a novel adjusted intervention. Lastly, while this study examined independent seniors functioning in an urban community, 22 of the 24 participants were women. This demographic suggests that the generalizability of the findings may be limited to women.

We encourage future research to incorporate an additional measurement time, six months post-Time 2. This extended measurement time would help better ascertain to what extent the reported changes are internalized^46^. We posit that, with time, the differences between the intervention and control groups would be maintained and even strengthened, especially if the participants continued practicing mindfulness until the third measurement time independently.

In summary, a brief mindfulness intervention diminished levels of worry among elderly participants, as found in earlier studies^47^. In addition, to our knowledge, this is the first study to suggest that a mindfulness intervention improves attitudes toward seeking mental health treatment among the elderly. These findings have direct implications for seniors' quality of life and well-being, especially since mindfulness exercises can be practiced at any time, do not require accessories, and are independent of one's physical state. Furthermore, due to mindfulness practice, more seniors will likely allow themselves to utilize mental health services. It is important to note, however, that the mindfulness intervention did not diminish anxiety and, likely due to a "floor effect," did not significantly improve measures of depression. The current study encourages mindfulness-based interventions for seniors (MBIS), which can be taught at low cost in group settings, at community centers or in public health clinics. Providing these interventions in nursing homes may offer a critical solution and great potential for increasing participation among older adults.

## Supplementary Information

Supplementary Information.
