# Supplementary material for: The effects of mindfulness meditation versus CBT for anxiety on emotional distress and attitudes toward seeking mental health treatment: a semi-randomized trial
Source: Sci Rep. 2022 Nov 16;12:19711. doi: 10.1038/s41598-022-24256-9 (PMC9668224; doi:10.1038/s41598-022-24256-9)
Supplement: Supplementary file 1 — Supplementary Information. [file 41598_2022_24256_MOESM1_ESM.docx]

**Appendices**

Appendix 1 - Complete MBIS Intervention

Session 1 Introduction to Mindfulness

10 Minutes Rules + Motivation

5 Minutes Attention to Breathing Exercise

5 Minutes Sharing

5 Minutes Conscious Eating Exercise

5 Minutes Home Assignments

Session 2 Body Scan

10 Minutes Introduction and Sharing

10 Minutes Body Scan Exercise

5 Minutes Sharing

5 Minutes Explanation of Home Assignments

Session 3 Gentle and non-Judgmental Attention, Identification of Automatic Patterns

5 Minutes Introduction and Sharing

5 Minutes Awareness of Breathing

5 Minutes Body Scan

5 Minutes Exercise: Mindfulness to Voices and Thoughts - Short

5 Minutes Sharing

5 Minutes Explanation of Home Assignments

Session 4 My Thoughts are Not Facts

5 Minutes Exercise: Mindfulness to Voices and Thoughts

5 Minutes Sharing and Explanation

5 Minutes Explanation of “Stop” Exercise

10 Minutes Exercise Breathing Space in Three Steps

5 Minutes Explanation of Home Assignments

Session 5 What We Object To Increases, What We Accept Decreases

10 Minutes Attention to Feelings of Fatigue/Tension/Irritation

5 Minutes Sharing

5 Minutes Exercise Breathing Space

10 Minutes Explanation of Home Assignments

Session 6 Like a Mountain

10 Minutes Sharing and Questions

2 Minutes Introduction to Exercise

10 Minutes Exercise “Like a Mountain”

3 Minutes Summary of Exercise

5 Minutes Explanation of Home Assignments

Session 7 Coping with Tension Arousing Thoughts

10 Minutes Sharing and Introduction to Exercise

6 Minutes Exercise: Mindfulness to Tension Arousing Thought or Emotion

4 Minutes Sharing

5 Minutes Abbreviated “Like a Mountain”

5 Minutes Summary and Explanation of Home Assignments

Session 8 Summary and What’s Next?

10 Minutes Introduction

10 Minutes Summary According to the Handouts + “What’s Next?”

10 Minutes Exercise “Like a Mountain”

Appendix 2 – Complete CBT Intervention

Session 1 Introduction

10 Minutes Rules + Motivation

5 Minutes Explanation of Diaphragmatic Breathing

5 Minutes Exercise Diaphragmaitc Breathing

10 Minutes Explanation of the Importance in Setting Aims for Change and Explanation of Home Assignments and their Importance

Session 2 Automatic Thoughts

10 Minutes Conversation About Aims for Change and Life Goals (Preparing a List + Ranking (the various components of the list) with the Group)

10 Minutes Explanation of Automatic Thoughts

5 Minutes Diaphragmatic Breathing

5 Minutes Explanation of Home Assignments

Session 3 Monitoring a Situation

20 Minutes Explanation of the First Part of the Table (Situation, Thought, Emotion) + Sharing of Home Assignment

5 Minutes Diaphragmatic Breathing

5 Minutes Explanation of Home Assignment

Session 4 Biases in Thinking

5 Minutes Sharing of Home Assignment (Problems, Questions)

15 Minutes Explanation of Biases in Thinking + Explanations + Handouts

5 Minutes Diaphragmatic Breathing

5 Minutes Explanation of Home Assignments

Session 5 Biases in Thinking

20 Minutes “Classic” Case in Order to Show How to Fill out The Table of the Situation + Automatic Thought + Emotion + Bias in Thinking

5 Minutes Diaphragmatic Breathing

5 Minutes Explanation of Home Assignment

Session 6 Flexible Thinking

20 Minutes Explanation of Modifying Thinking + Example According to a Case

5 Minutes Diaphragmatic Breathing

5 Minutes Explanation of Home Assignment

Session 7 Flexible Thinking

20 Minutes Sharing of Home Assignment + Answering Questions and Review of Important Information, According to Need

5 Minutes Diaphragmatic Breathing

5 Minutes Explanation of Home Assignment

Session 8 Summary Session

7 Minutes Summary of My Findings – So What Did We Even Do?

10 Minutes Feedback and Joint Discussion, “What Did You Take From the Course?” “What Do You Think You’ll Do The Next Time You’ll Find Yourself in an Uncomfortable Situation?”

8 Minutes Summary Exercise Using a Classic Example – The Participants Will Help Complete the Row in the Table From Beginning Till the End

5 Minutes Diaphragmatic Breathing
